# Using a computational model to quantify the potential impact of changing the placement of healthy beverages in stores as an intervention to “Nudge” adolescent behavior choice

**DOI:** 10.1186/s12889-015-2626-0

**Published:** 2015-12-23

**Authors:** Michelle S. Wong, Claudia Nau, Anna Yevgenyevna Kharmats, Gabriela Milhassi Vedovato, Lawrence J. Cheskin, Joel Gittelsohn, Bruce Y. Lee

**Affiliations:** Global Obesity Prevention Center (GOPC) and Johns Hopkins, Johns Hopkins School of Public Health, 615 N. Wolfe St, Baltimore, MD 21205 USA; Department of Health Policy and Management, Johns Hopkins School of Public Health, 624 N. Broadway, Baltimore, MD 21205 USA; Department of International Health, Johns Hopkins School of Public Health, 615 N. Wolfe St, Baltimore, MD 21205 USA; Health and Society Institute, Baixada Santista Campus, Federal University of Sao Paulo, 136 Silva Jardim Street, Santos, SP 11015-020 Brazil; Johns Hopkins Weight Management Center, Johns Hopkins School of Public Health, 550 N. Broadway,Suite 1001, Baltimore, MD 21205 USA; Department of Health, Behavior & Society, Johns Hopkins Weight Management Center, Johns Hopkins School of Public Health, 615 N. Wolfe St, Baltimore, MD 21205 USA; Department of International Health, Center for Human Nutrition, Johns Hopkins School of Public Health, 615 N. Wolfe St, Baltimore, MD 21205 USA

**Keywords:** Obesity, Corner store intervention, Sugar sweetened beverage consumption

## Abstract

**Background:**

Product placement influences consumer choices in retail stores. While sugar sweetened beverage (SSB) manufacturers expend considerable effort and resources to determine how product placement may increase SSB purchases, the information is proprietary and not available to the public health and research community. This study aims to quantify the effect of non-SSB product placement in corner stores on adolescent beverage purchasing behavior. Corner stores are small privately owned retail stores that are important beverage providers in low-income neighborhoods – where adolescents have higher rates of obesity.

**Methods:**

Using data from a community-based survey in Baltimore and parameters from the marketing literature, we developed a decision-analytic model to simulate and quantify how placement of healthy beverage (placement in beverage cooler closest to entrance, distance from back of the store, and vertical placement within each cooler) affects the probability of adolescents purchasing non-SSBs.

**Results:**

In our simulation, non-SSB purchases were 2.8 times higher when placed in the “optimal location” – on the second or third shelves of the front cooler – compared to the worst location on the bottom shelf of the cooler farthest from the entrance. Based on our model results and survey data, we project that moving non-SSBs from the worst to the optional location would result in approximately 5.2 million more non-SSBs purchased by Baltimore adolescents annually.

**Conclusions:**

Our study is the first to quantify the potential impact of changing placement of beverages in corner stores. Our findings suggest that this could be a low-cost, yet impactful strategy to nudge this population—highly susceptible to obesity—towards healthier beverage decisions.

## Background

While manufacturers, particularly sugar sweetened-beverage (SSB) companies, expend considerable amount of effort, time, and resources determining where to place their products in stores to maximize purchasing [1], most of this research is proprietary and unavailable to the broader research community. Public health could potentially take advantage of product placement to curb SSB consumption, which is linked to obesity risk [2]. This strategy of product placement takes advantage of the fact that consumers may subconsciously rely on environmental cues – such as visibility, accessibility, and convenience – to help guide their purchasing decisions [3, 4]. Placing healthier beverage options in more visible and easily accessible locations, public health can potentially “nudge” consumers away from SSBs without limiting their range of beverage choices by making these healthier choices more appealing [4].

This study aims to quantify the effect of non-SSB product placement in corner stores on adolescent beverage purchasing behavior. We developed a computational simulation model to address this. “Nudging” strategies, including product placement, tend to be relatively simple and easy to implement [4], making them ideal for settings with limited resources, such as corner stores.

## Methods

We developed a decision-analytic computer simulation model in TreeAgePro 2014 (TreeAge Pro, Williamstown, MA) to quantify the impact of changing non-SSB (e.g., bottled water) placement in corner stores on adolescent non-SSB purchases. Prior research suggests that the effect of marketing strategies, such as product placement, varies by consumer preferences [5]. Product placement strategies include placement in the first cooler, vertical placement within a cooler, and horizontal distance from the back of the store [6–8].

**Fig. 1:**
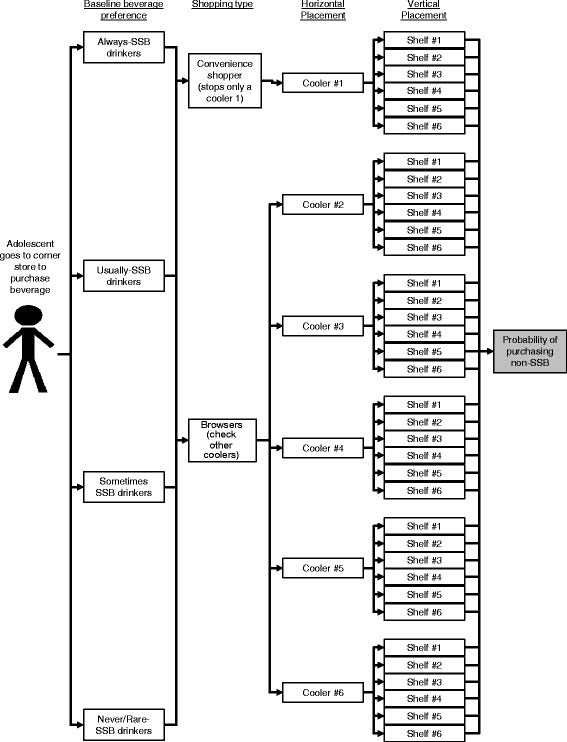
Model structure

Figure 1 displays the model structure. Table 1 provides input parameters and data sources. We modeled 6 coolers, each with 6 shelves, which are labeled numerically by location (cooler 1 was closest to the entrance, and shelf 1 was the top). Information about cooler location came from an assessment of 29 corner stores conducted by the *B’more Healthy Communities for Kids* (BHCK) program, an obesity prevention program in Baltimore, Maryland [9]. More information about the BHCK program is available from in Gittelsohn et al. (2014) [9]. Information about cooler width came from online searches of retail beverage coolers dimensions [10].

**Table 1:** Model Input parameters and values

Parameter	Probability of non-SSB purchase	Source
**Base preference Probability**		
Always SSB	0	[9]
Usually SSB	0 – 28.4	[9]
Sometimes SSB	28. 5 – 54.4	[9]
Rare/never SSB	>54.4	[9]
**“Convenience shopper” Probability**		
Baseline “convenience shopper”	32.6	[11]
Loyalty		
Always SSB	27.0	[5]
Usually SSB	75.0	[5]
Sometimes SSB	100.0	[5]
Rare/never SSB	27.0	[5]
**Horizontal Effect** ^b^		
Cooler 1	17.9	[7]
Cooler 2	17.4	[7]
Cooler 3	16.9	[7]
Cooler 4	16.4	[7]
Cooler 5	15.9	[7]
Cooler 6 (back)	15.4	[7]
**Vertical Effect** ^b^		
Shelf 1 (top)	17.0	[7]
Shelf 2	17.2	[7]
Shelf 3	17.1	[7]
Shelf 4	16.8	[7]
Shelf 5	16.3	[7]
Shelf 6	15.5	[7]

Notes:

^a^Probabilities of being a convenience shopper were calculated as the product of the baseline probability of being a convenience shopper and the probabilities associated with brand loyalty for each type of SSB drinker

^b^Horizontal and Vertical effects were calculated based upon regression coefficient parameters obtained from [7] and average retail beverage cooler dimensions

Adolescents are assigned to one of four beverage preference groups based on their probability of purchasing an SSB: always, usually, sometimes, rarely/never. This data came from beverage purchasing data from BHKC’s sample of 211 predominantly low-income African American adolescents, ages 10 to 14, who purchased at least one beverage from a corner store during the prior week. Preference groups were assigned based on quartiles of the proportion of non-SSB beverages purchased.

Based on baseline preference, individuals are further classified into “convenience shoppers” who only walk to the first cooler (cooler 1), or “browsers” who also check the remaining coolers (cooler 2 – 6). We assume that “always” and “never SSB” drinkers are more likely to be browsers than “sometime” or “usually SSB” drinkers. The probability of being a browser or convenience shopper came from a study that assessed the effect of food order in a buffet line on diner food selection [11]. We vary this probability by a beverage-preference-specific “loyalty” parameter, obtained from data on the effect of consumer brand loyalty on brand-switching behavior [5].

All individuals are assumed to be influenced by a “vertical effect”: distance to the middle and bottom shelf. Browsers are also influenced by the “horizontal effect” of distance to the back of the store. Vertical and horizontal effect parameters came from an observational study of product placement on canned soups sales [7]. We assumed that these effects would be similar for a range of products, including beverages.

We assume that adolescents only purchase one beverage per store visit; and beverages are the same price and size and have the same number of facings (i.e., amount of shelf space allotted to a product). Based on CDC’s definition, non-SSBs include: water, diet soda and soft drinks, 100 % fruit juice, and milk, while SSBs include non-diet soft drinks/sodas, sweetened fruit juices, and caloric sports drinks [12].

We simulate purchases by 1000 individuals. We calculated the probability of purchase for each location relative to the “worst” location: shelf 6 (bottom shelf) of cooler 6 (farthest from entrance), and stratified results by baseline preference.

### Sensitivity analyses

Since our model used particular assumptions and parameters, we conducted a comprehensive set of sensitivity analyses to test the robustness of our results to changing circumstances, assumptions, and variability. Sensitivity analyses varied beverage preference, the baseline probability of being a “convenience shopper” vs. “browser”, and the “horizontal” and “vertical” effects. We tested the following five scenarios: 1) increasing the probability that the “always SSB” group purchased a non-SSB up to 25 %, 2) doubling the probability of being a “convenience shopper”, 3) halving the probability of being a “convenience shopper, 4) doubling the “horizontal” and “vertical” effects simultaneously (i.e., doubling the difference in the probability of purchase between coolers/shelves), and 5) halving the “horizontal” and “vertical” effects simultaneously (i.e., halving the difference in the probability of purchase between coolers/shelves).

## Results

**Fig. 2:**
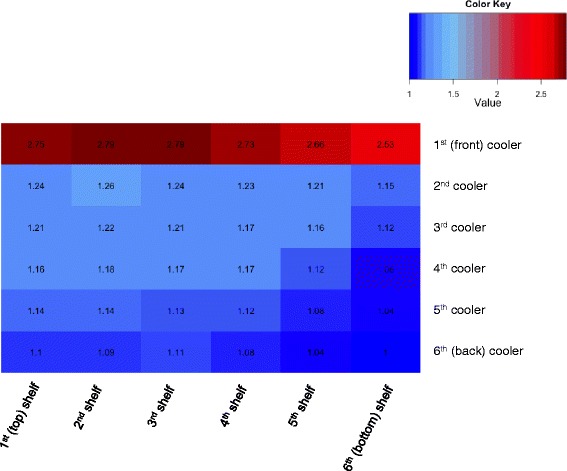
Heat map of the probability of purchasing a non-SSB by beverage placement

Figure 2 shows the probability of purchasing a non-SSB for each shelf of each cooler relative to the “worst” location (cooler 6, shelf 6). Our model indicates that the optimal location was in cooler 1 (closest to the store entrance), on shelf 2 and 3 (at approximately eye level). A non-SSB placed here was 2.8 times more likely to be purchased compared to the worst location. A non-SSB placed on any shelf in the first cooler was more likely to be purchased than in coolers 2 – 6. Even on the bottom shelf of the first cooler, a non-SSB is projected to be 2.5 times more likely to be purchased compared to the worst location. Among the remaining coolers 2 through 6, cooler 2, shelf 2 was the second most optimal location (1.3 times greater than worst location).

**Fig. 3:**
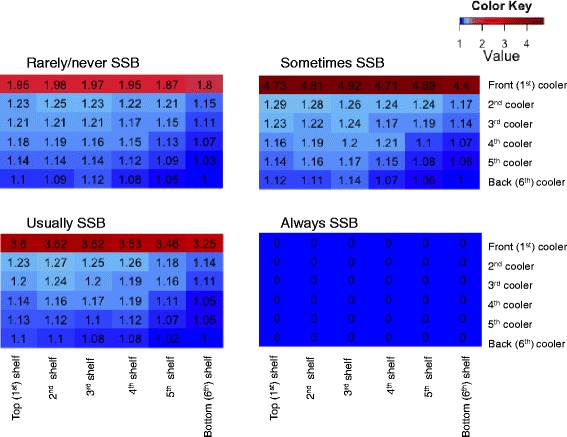
Heat map of the probability of purchasing a non-SSB by beverage placement stratified by beverage placement

Figure 3 stratifies the relative probability of purchase by beverage preference. The effect of product placement was strongest among “sometimes SSB” drinkers: they were 4.9 times more likely to purchase non-SSBs in the optimal location compared to the worst location. While product placement had a modest effect on “usually SSB” and “rarely/never SSB” drinkers, we found no effect among “always SSB” drinkers.

Assuming that beverage preferences and purchases from the BHCK sample are representative of Baltimore’s adolescent population [13], we extrapolate our findings to project the increase in non-SSBs purchased by all Baltimore adolescent over the course of a year under 3 scenarios: moving non-SSBs from the worst to optimal position; moving non-SSBs from the worst to second most optional location; and moving non-SSBs from the second most optional to optimal location. We project that the first scenario would result 5.2 million more non-SSBs, Scenario 2 would result in an approximate 700,000 more non-SSBs, and scenario 3 would result in 366,000 more non-SSBs purchased by Baltimore adolescents.

Sensitivity analyses suggested that our results are overall robust to potential mismeasurement of preference and shopping behaviors. Sensitivity analysis results are show in Table 2. Cooler 1, shelf 2 and 3 remained the optimal positions, but the relative magnitude of increases in non-SSB purchases changed slightly. While our model appeared to be most sensitive to changes in the convenience shopper parameter, results remained consistent and within the same order of magnitude as our original results (Fig. 2). For example, even when we halved the “convenience shopper” parameter, adolescents are still nearly twice as likely to purchase a non-SSB in the optimal location compared to the worst location. Changes the other parameters (“always SSB” probability, and horizontal and vertical effects) resulted in a range of relative probabilities of purchase from 2.5 to 3.5 in the optimal location (cool 1, shelf 2 or 3) compared to worst location.

**Table 2:** Sensitivity analysis parameters and results

Variable	Probability				Relative probability of purchase in the optimal location compared to the worst location^a^
Convenience shopper	18.3 (half)				1.97
	65.2 (double)				5.01
Always SSB	25				3.50
Horizontal and Vertical effects	Vertical (double)		Horizontal (double)		3.47
	Shelf 1 (top)	17.4	Cooler 1	19.2	
	Shelf 2	17.8	Cooler 2	18.2	
	Shelf 3	17.6	Cooler 3	17.2	
	Shelf 4	17.0	Cooler 4	16.2	
	Shelf 5	16.0	Cooler 5	15.2	
	Shelf 6	14.4	Cooler 6 (back)	14.2	
	Vertical (half)		Horizontal (half)		2.51
	Shelf 1 (top)	17.1	Cooler 1	17.3	
	Shelf 2	16.9	Cooler 2	17.0	
	Shelf 3	16.9	Cooler 3	16.8	
	Shelf 4	16.7	Cooler 4	16.5	
	Shelf 5	16.5	Cooler 5	16.3	
	Shelf 6	16.1	Cooler 6 (back)	16.0	

Note: ^a^Optimal location: cooler 1, shelf 2 or 3; relative to worst location: cooler 6, shelf 6

## Discussion

Our study assessed product placement as public health strategy to improve beverage purchases. It is the first to quantify the potential impact of changing non-SSB placement in corner stores and suggests that this could be an effective strategy for populations highly susceptible to childhood and adolescent obesity. The strongest effect occurred among individuals who sometimes drank SSBs, suggesting that this nudging technique might be most effective in preventing weight gain in a population who might be at the verge of developing this unhealthy habit or are considering changing bad habits. Our results do not suggest that product placement should be the only interventions. For example, those who always drink SSBs will need other public health strategies (e.g., education, soda tax) to alter their beverage preferences before nudging interventions can be effective.

While the parameters and assumptions of our model came from studies that were not conducted in SSBs or other sugary foods that may potentially be habit-forming [14], we conducted a variety of sensitivity analyses to assess how changes to these parameters and assumptions might alter our results. Although the relative probabilities of purchased changed in our sensitivity analyses, these results were still within a similar order of magnitude to our baseline model. To our knowledge, data on SSBs or other similar sugary products that can be used to parameterize our model does not exist, but our sensitivity analyses gives us confidence that we would observe similar results. Although our model may overestimate the effects of product placement on non-SSB purchases for some portion of the population – particularly “always” and “usually SSB” drinkers who may be more willing to seek out SSBs – our findings will still apply to the majority of the population who only occasionally drinks SSBs [15]. Additionally, other studies have found that product placement, such as the physical distance from a serving bowl, or placement by the cash register, affects consumption of sugary snack foods that may be similarly habit forming, including M&Ms® [8], cereal bars [16], and animal crackers [17]. This suggests that product placement does affect consumption behavior across a variety of products, including sugary, habit-forming products.

Some corner stores may have cooler space limitations (e.g., beverage suppliers may purchase or reserve optimal cooler space), and certain placement locations may not be available. We present our results in the form of a heat map, a simple visual tool that shows the effect of product placement for the entire cooler space. This heat map will allow corner store owners and other interested stakeholders, such as public health researchers and policy makers, to identify possible non-SSB product placement locations given a corner store’s specific space constraints. Since store owners negotiate stocking based upon store profit, a successful product placement intervention should increase demand for non-SSBs, which in turn, may encourage store owners to both place non-SSBs in more easily accessible locations and increase the stocking of non-SSBs. Additionally, understanding the potential of product placement on healthier beverage consumption is valuable to a variety of stakeholders, such as public health researchers, and can help inform other interventions that take advantage of marketing tools and nudging techniques.

While computational models are simplifications of real life and may not account for all of the factors that may affect purchasing decisions (e.g., beverage prices and sizes, number of beverage facings, familiarity with beverages over time) [8, 14, 16, 17], they serve as method to merge disparate data from various sources to test hypotheses that may take considerable time, effort, and resources to test in natural settings. Many industries including advertisers and marketers utilize computational models to plan their strategies before executing them. A computational model is particularly apt to assess the potential of this intervention in a corner store setting given their limited technological and human resources, which would make conducting an evaluation challenging and with limited generalizability. Results from our computational model should be used in conjunction with and to help inform future quasi-experimental studies or intervention evaluation.

## Conclusions

Our model suggests that placing non-SSBs at the front of the store, at approximately eye level, may “nudge” individuals, particularly those who occasionally drink SSBs towards healthier beverage choice. These results are an initial step in exploring product placement’s potential and can guide the future implementation of this low-cost public health intervention.
